# Diversity and robustness of bone morphogenetic protein pattern formation

**DOI:** 10.1242/dev.192344

**Published:** 2021-04-01

**Authors:** Aasakiran Madamanchi, Mary C. Mullins, David M. Umulis

**Affiliations:** 1 Agricultural and Biological Engineering. Purdue University, West Lafayette, IN 47907, USA; 2 Polytechnic Institute, Purdue University, West Lafayette, IN 47907, USA; 3 Department of Cell and Developmental Biology, University of Pennsylvania Perelman School of Medicine, Philadelphia, PA 19104, USA; 4 Weldon School of Biomedical Engineering, Purdue University, West Lafayette, IN 47907, USA

**Keywords:** Morphogen gradient, BMP patterning, Zebrafish, *Drosophila*, Axis formation

## Abstract

Pattern formation by bone morphogenetic proteins (BMPs) demonstrates remarkable plasticity and utility in several contexts, such as early embryonic development, tissue patterning and the maintenance of stem cell niches. BMPs pattern tissues over many temporal and spatial scales: BMP gradients as short as 1-2 cell diameters maintain the stem cell niche of the *Drosophila* germarium over a 24-h cycle, and BMP gradients of several hundred microns establish dorsal-ventral tissue specification in *Drosophila*, zebrafish and *Xenopus* embryos in timescales between 30 min and several hours. The mechanisms that shape BMP signaling gradients are also incredibly diverse. Although ligand diffusion plays a dominant role in forming the gradient, a cast of diffusible and non-diffusible regulators modulate gradient formation and confer robustness, including scale invariance and adaptability to perturbations in gene expression and growth. In this Review, we document the diverse ways that BMP gradients are formed and refined, and we identify the core principles that they share to achieve reliable performance.

## Abstract (precis)

**Summary:** This Review looks at BMP morphogen gradient patterning in diverse developmental contexts including in *Tribolium*, *Drosophila*, *Xenopus* and zebrafish early embryos, as well as the *Drosophila* wing imaginal disc and germarium.

## Introduction

The core bone morphogenetic protein (BMP) signaling components are largely conserved across metazoans (reviewed by Carroll et al., 2005; De Robertis, 2008). In vertebrates and invertebrates alike, the BMP signaling cascade is initiated with the binding of an extracellular ligand dimer to a heterotetrameric transmembrane receptor complex. The ligand-activated receptor complex consists of two Type I and two Type II serine/threonine kinase receptors. Ligand-bound Type II receptors phosphorylate the intracellular GS domain of associated Type I receptors. In turn, phosphorylated Type I receptors initiate recruitment and phosphorylation of BMP pathway-specific receptor-regulated SMADs (R-SMADs) (reviewed by Bandyopadhyay et al., 2013). Phosphorylated R-SMADS bind a co-SMAD to form a cytoplasmic complex with altered nucleo-cytoplasmic shuttling properties; this complex accumulates in the nucleus, where it functions with other transcription factors to regulate downstream gene expression (Schmierer et al., 2008).

Vertebrate BMP pathway components are often highly redundant and can include multiple homologs of each signaling component because of genome-wide duplications and expansion events. For example, more than 20 BMP ligands have been identified in vertebrate species, comprising two major ligand classes that are represented by three genes in *Drosophila*. These ligand-class genes in vertebrates are *BMP2/4* and *BMP5/6/7/8* (reviewed by Miyazono et al., 2019; Zinski et al., 2018). However, there is increasing evidence that ligand heterodimer signaling is crucial in several developmental contexts (Kim et al., 2019; Little and Mullins, 2009; Valera et al., 2010). In addition, two subtypes each of Type I and Type II receptors, and multiple R-SMAD proteins, have been identified (reviewed by Brazil et al., 2015). Diversity in canonical signaling systems allows combinatorial signal processing and regulatory flexibility that is considered to be crucial for adaptation to specific contexts (Antebi et al., 2017; Llimargas and Lawrence, 2001; reviewed by Mueller and Nickel, 2012). However, even BMP signaling systems with relatively few components exhibit context-specific patterning processes with highly divergent spatial and temporal characteristics.

**Fig. 1.**
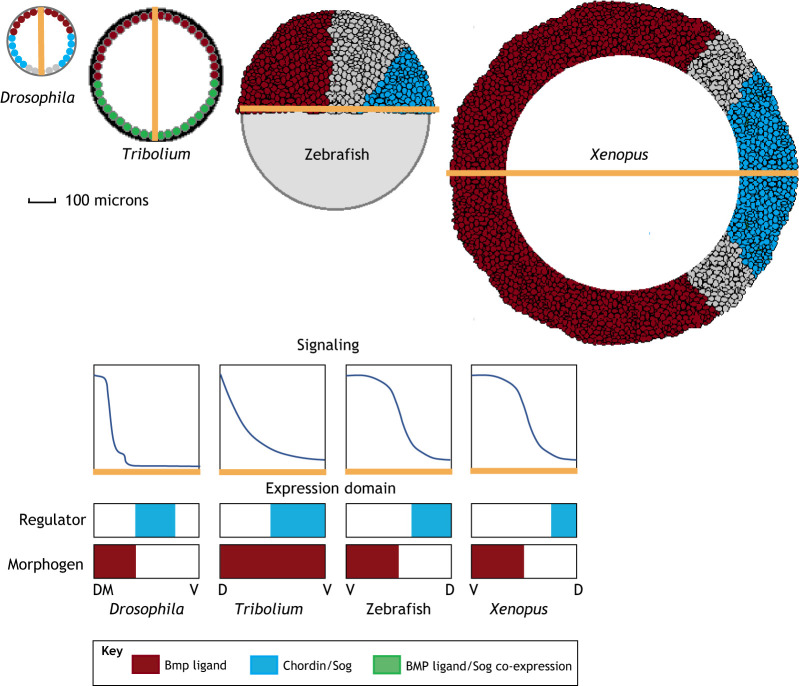
**Signaling gradient profiles and expression domains of BMP patterned embryos.** Top schematics show Dpp and Sog expression domains in the *Drosophila* embryo (240 μm; transverse section). Dpp expression domain and co-expression of Dpp and Sog in the *Tribolium castaneum* embryo (480 μm; transverse section). Expression domains of *bmp* ventrally and *chordin* dorsally in the zebrafish embryo (700 μm; late blastula) and the *Xenopus* embryo (1.2 mm; early gastrula, transverse section). Lower schematics show qualitative graphs of BMP signaling gradients and the expression domains for the morphogen and negative regulator. The yellow *x*-axis in the signaling graphs corresponds to the yellow line in the images of the top schematic. D, dorsal; DM, dorsal midline; V, ventral.

The BMP morphogen gradient manifests in a diversity of forms in different developmental niches and across species, revealing a remarkable capacity to operate at different length scales, from controlling cells near the source over distances of 5-10 μm, to patterning over long distances exceeding 0.5 mm in length (Fig. 1). The ways in which the gradient is formed between these systems also represents incredible diversity.

BMP signaling in patterning in *Drosophila* has been well studied in various developmental contexts. In *Drosophila*, the BMP ligands include the BMP2/4 ortholog, Decapentaplegic (Dpp), and the BMP5/6/7/8 orthologs, Screw (Scw) and Glass bottom boat (Gbb). The receptor portfolio is similarly limited, with only two Type I receptors, Thickveins (Tkv) and Saxophone, and two Type II receptors, Punt (Put) and Wishful thinking (Wit). Downstream Dpp/BMP signaling in *Drosophila* is transmitted through a single R-Smad, Mothers against Dpp (Mad), and a single co-Smad, Medea (reviewed by Hamaratoglu et al., 2014). Despite this relative simplicity, *Drosophila* Dpp/BMP signaling drives vastly different patterning profiles in different organs, such as the ovarian germarium, embryos, the wing imaginal disc and cross-vein formation in developing pupal wings. For example, in the *Drosophila* germarium, BMPs are secreted from a localized source and only extend a few cell diameters into the organ, whereas in development of embryos, wing discs and pupal wings, Dpp has a much broader range and diversity of how the gradients are formed, including gradients that expand, gradients that contract and gradients that pattern regions orthogonal to the source.

In other species, BMPs play a similarly broad role in patterning multiple tissues at different scales, including during the earliest stages of axis development in other insects, such as the flour beetle *Tribolium*, and a number of vertebrate models including zebrafish and *Xenopus*. Each of these diverse contexts poses different constraints on gradient formation, including domain size, time for patterning, rate of feedback, and requirements for robustness in scale, temperature variation and genetic perturbations. In this Review, we introduce general principles for how the BMP gradients are formed and then discuss how each biological context constructs a gradient using these mechanisms in different ways. We explore how extracellular regulatory machinery operates within these systems to shape BMP signaling across diverse developmental contexts and we discuss how the machinery, when coupled with feedback, provides the emergent properties of robustness and scaling in many contexts. We begin by describing Dpp/BMP gradient formation in the *Drosophila* germarium and wing imaginal disc, in which ligands are regulated by many non-diffusing regulatory molecules that control gradient range. We then extend our analysis to systems that combine diffusible and non-diffusible regulators that control BMP signaling in *Drosophila* pupal wings and in the embryos of *Drosophila*, *Tribolium*, zebrafish and *Xenopus*.

## Forming BMP gradients

**Fig. 2.**
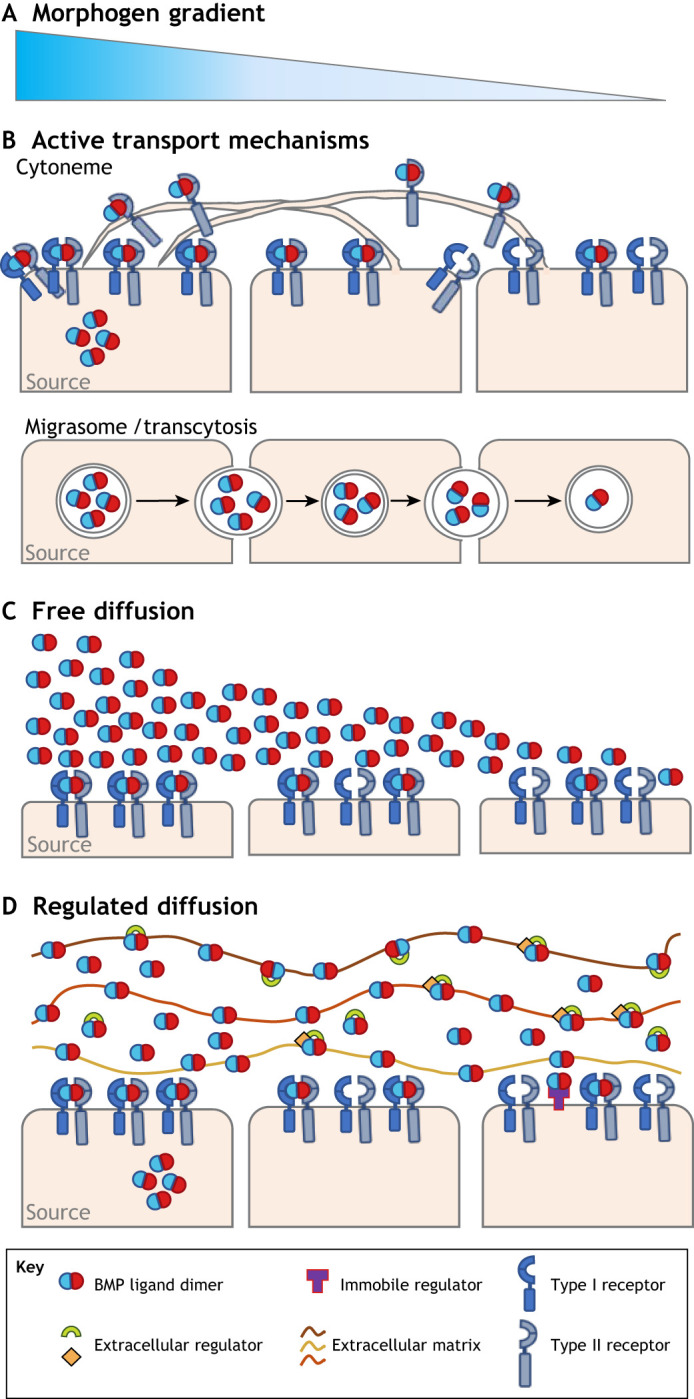
**Morphogen gradient formation mechanisms.** (A) Morphogen gradient concept. (B) Gradient formation via active transport mechanisms: cytonemes (top) and migrasome/transcytosis (bottom). Cytonemes are cellular projections which can emanate from cells towards the morphogen source. Cytonemes carry Type II Bmp receptors which can take ligand back to the cell where they can signal in a receptor complex. Migrasome/transcytosis shows vesicle-based transport of ligand away from the source. (C) Gradient formation by free (passive) diffusion. Ligand diffuses from areas of high concentration near the source to areas of lower concentration. Pre-steady state ligand concentration is depicted. (D) Gradient formation via regulated diffusion. Extracellular matrix, immobile regulators and diffusible extracellular regulators all act to regulate diffusion. Note: receptors and ligands not to scale.

During development, BMP ligands function as morphogens and direct the patterning and organization of tissues through interpretation of its concentration gradient (Fig. 2A). Three general classes of gradient formation mechanisms have emerged: active transport, free diffusion and regulated diffusion (Fig. 2B-D), wherein extracellular binding molecules interact with the ligand to change its ultimate distribution (reviewed by Müller et al., 2013; Teleman et al., 2001). There are at least two mechanisms of active transport: vesicle-based transport, including transcytosis and migrasomes, in which morphogen ligands are shuttled across tissue via repeated cycles of receptor-mediated endo- and exocytosis (reviewed by Erban and Othmer, 2014; González-Gaitán and Jäckle, 1999; Greco et al., 2001; Jiang et al., 2019; Kicheva et al., 2007; Othmer et al., 1988; Panáková et al., 2005; reviewed by Restrepo et al., 2014), and cytoneme-mediated transport, in which extensive actin-based filopodial networks act as direct conduits for morphogen transmission to target cells (Ramírez-Weber and Kornberg, 1999). Other non-directional forms of active transport, such as transcytosis or transport on microtubule-based motor proteins, can be modeled mathematically as diffusion-like (reviewed by Bollenbach et al., 2005; Thompson et al., 2018).

Regulated diffusion and free diffusion mechanisms represent the largest classes of mechanisms that occur during BMP-mediated embryo development across taxa. Within these classes, it is helpful to distinguish between regulated diffusion by immobile factors, such as receptors and co-receptors, extracellular matrix (ECM) components and other immobile binders that impact diffusion range between being completely free with a long range or being hindered and having a short range. This framework is particularly helpful in understanding the *Drosophila* germarium and wing disc. Regulated diffusion mechanisms also include those that rely on a bevy of diffusible secreted molecules that bind to the BMP ligands and slow, speed up, or even change the net transport of ligands throughout the embryo or tissue. Simulation of reaction-diffusion mathematical models of the transport and reaction steps of all the molecules is becoming more widely used to better understand the mechanisms and relative contributions of mobile and immobile factors on gradient formation.

The relative importance of these factors and mechanisms in gradient formation remains a topic of intense debate (reviewed by Akiyama and Gibson, 2015b; Lander et al., 2002; Lo et al., 2015; Müller et al., 2013; Zhou et al., 2012). Reaction-diffusion mathematical models, which are distinct to Turing's reaction-diffusion mechanism of patterning, can be used to describe the spatiotemporal dynamics of BMP in terms of experimentally observable biophysical rates (Box 1). Integrating quantitative biophysical experiments with mathematical modeling provides a rigorous approach to test the plausibility of hypothesized mechanisms guiding pattern formation (reviewed by Thompson et al., 2018). Importantly, analysis through a reaction-diffusion modeling framework can remain somewhat agnostic of the class of gradient formation and can account for differences between these mechanisms (Box 1). Simulation also now serves as an additional means to study the interrelatedness of the patterning mechanisms and provides a way to interpret how minor changes between systems that share components can achieve incredible diversity in how patterns of BMP signaling are formed and maintained.

**Box 1. Reaction-diffusion model framework**

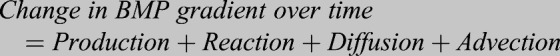

Within this framework, reviewed at length in Thompson et al. (2018), the ‘Production’ term reflects both the spatial extent of the ligand expression domain, as well as the rate of ligand production. Clearance of BMP ligand via receptor-mediated internalization and interactions with extracellular regulators are accounted for within the ‘Reaction’ term. The ‘Diffusion’ term refers to the movement of the morphogen through extracellular space via random walk, and accounts for both the rate of diffusion and the amount of ligand diffusing. Binding reactions with a diverse set of extracellular molecules can influence the effective diffusion of the morphogen; interactions with both immobile molecules, including Type IV Collagen and other extracellular matrix components (Box 2), as well as with mobile molecules such as Chordin or Sog, can have the effect of concentrating or dispersing the morphogen, depending on the biophysical parameters involved. Finally, the ‘Advection’ term reflects active transport forms of BMP morphogen movement (e.g. bulk cell movement as a tissue expands), or molecular transport mediated by biological processes that are directional and independent of ligand diffusion (e.g. cytoneme-mediated transport; Fig. 2B).

**Box 2. Influence of immobile regulators on morphogen diffusion**

The effect of immobile regulators on morphogen diffusion can be illustrated through the mathematics of reaction-diffusion as demonstrated below:(1)
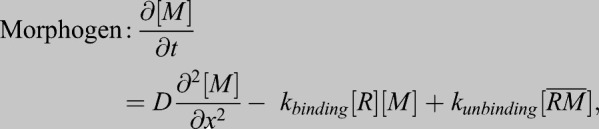
(2)
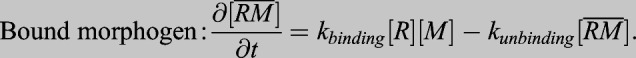

[*M*] is morphogen concentration, 
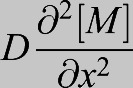
 is morphogen diffusivity, *x* is distance from source, [*R*] is concentration of immobile regulator and 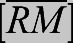 is concentration of morphogen bound to regulator. The rate constants for binding and unbinding of regulator and morphogen are *k*_*binding*_ and *k*_*unbinding*_.

Summing Eqns 1 and 2 for an effective equation for the total morphogen in the systems gives:(3)
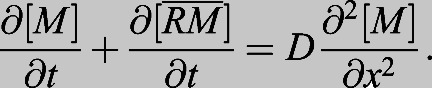
 If regulators are abundant, that is 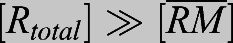 as is often the case, and morphogen-regulator binding is faster than diffusion, free morphogen and bound morphogen are in local equilibrium with:
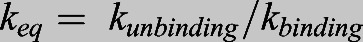
and
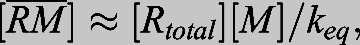

where *R*_*total*_ and *k_eq_* are constants.

Therefore, Eqn 3 can be rewritten as:(4)
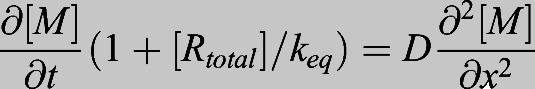

and further rearranged to give:(5)
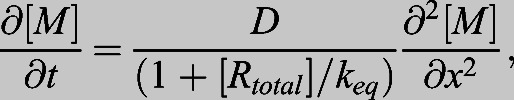

where greater regulator concentration, [*R*_*total*_], lowers effective diffusion and decreased regulator concentration causes greater effective diffusion. This generalized principle manifests in multiple contexts including in the *Drosophila* germarium, in which ablation of extracellular regulators expands the BMP gradient. The level of regulator, by this simple example, directly tunes the range of BMP distributions and can be modified for scale-invariance, robustness and gradient range over evolutionary time. A much more detailed look at this in the context of scaling is available in Čapek and Müller (2019), Umulis (2009) and Umulis and Othmer (2013).

## Shaping BMP gradients by immobile regulators

The similarities and differences in BMP signaling gradients provide great insight into how a common set of regulators make the pathway highly tunable for diverse contexts. We start by reviewing the *Drosophila* germarium and wing imaginal disc as illustrative examples of immobile regulators adapting the Dpp/BMP system to act on short and long distances, respectively.

### Tuning short-range BMP signaling in the *Drosophila* germarium

**Fig. 3.**
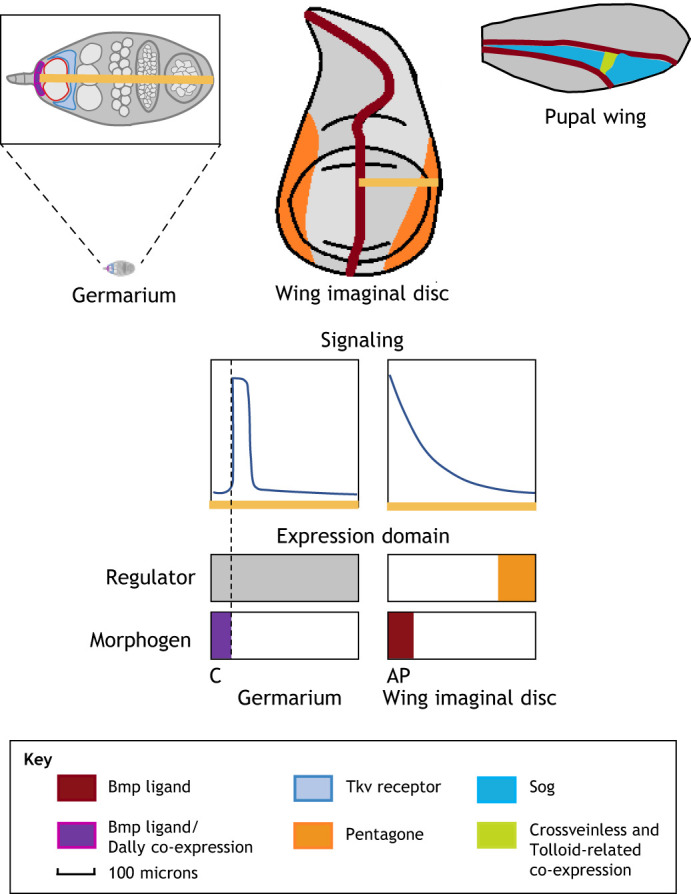
**Signaling gradient profiles and expression domains of BMP-patterned organs of different scales.** *Drosophila* germarium (top left): BMP/Dpp and Dally are co-expressed in cap cells (purple). Type IV Collagen Vkg (not pictured) is expressed throughout the GSC niche. Tkv is highly expressed in somatic escort cells. GSC cells are outlined in red (image modified from Sun et al., 2010). *Drosophila* third instar imaginal wing disc (top middle): BMP/Dpp is expressed in a narrow stripe at the AP boundary. Pentagone is expressed at the periphery. Crossvein formation in *Drosophila* pupal wing disc (top right): BMP/Dpp is expressed in longitudinal veins, Sog is expressed throughout the pupal wing. Crossveinless and Tolloid-related are expressed in the future posterior cross vein location where they can act to promote BMP/Dpp signaling by liberating ligand from Sog-Dpp complexes. Lower schematics show qualitative graphs of BMP signaling gradients and the expression domains for the morphogen and negative regulator. Germarium regulator depicted as a gray box to reflect ubiquitous presence of multiple regulators including Type IV Collagen Vkg. Signaling and expression domain graphs not shown for pupal wing disc as active transport does not take place over a single axis. AP, anterior posterior boundary; C, cap cells.

The *Drosophila* germarium, an ∼70-90 μm structure at the anterior end of the ovary, contains the germline stem cell (GSC) niche and is the site of oocyte production and differentiation. Dpp/BMP signaling is a crucial regulator of GSC recruitment and maintenance (Xie and Spradling, 1998), and germarium Dpp/BMP signaling is marked by tightly spatially-regulated short-range signaling (Song et al., 2004). Indeed, Dpp/BMP-induced receptor activation and downstream pMad signaling in GSCs is restricted to a single cell diameter (5 μm) of the Dpp/BMP-expressing cap cells (Michel et al., 2011; Xie and Spradling, 1998) (Fig. 3).This extreme spatial control is achieved through several mechanisms.

#### Extracellular regulators

Interactions with immobile ECM components help to localize Dpp/BMP ligands (Hayashi et al., 2009). For example, the HSPG protein Dally expressed in Dpp/BMP-producing cap cells, and the Type IV Collagen Vkg expressed within the GSC niche bind dynamically to Dpp/BMP and act to limit its diffusion range (Harris et al., 2011; Hayashi et al., 2009; Wang et al., 2008). In addition, high expression of the Type I Dpp/BMP receptor Tkv in the somatic escort cells surrounding the GSC niche provides additional spatial restrictions through a sink function as the receptor binds and sequesters excess Dpp/BMP (Luo et al., 2015). In each of these cases, an immobile extracellular regulator mediates changes in effective morphogen diffusivity and range (Fig. 3). A mathematical derivation is provided for the mechanistic basis for this phenomenon and also shows how tuning the non-diffusible regulators allows the gradient to have many different length scales (Box 2). Interestingly Dpp/BMP signaling in the germarium involves a Dpp/Gbb ligand heterodimer rather than the Dpp/Scw heterodimer in the dorsal blastoderm (Kawase et al., 2004). Understanding whether biophysical differences between Gbb and Scw in terms of diffusivity or Tkv affinity may have led to the selection of Gbb in the germarium presents an intriguing angle for further study. Alternatively, the involvement of Gbb here may represent subfunctionalization between these two ligands.

#### Adaptability of gradient range

To further refine the gradient, these spatial mechanisms are complemented by negative feedback mechanisms at the signal transduction level. Dpp/BMP signaling in the GSC niche promotes stem cell maintenance and directly represses expression of *bag of marbles* (*bam*), a translational repressor of key stem cell maintenance genes (Kawase et al., 2004). GSC daughter cells that are displaced from the niche receive less Dpp/BMP signaling and begin to undergo Bam-mediated differentiation into cystoblasts. In transitional cystoblasts, the translational regulator Brain tumor (Brat) establishes a bistable switch for differentiation by repressing *Mad* and thereby inhibiting Dpp/BMP signal transduction in these cells, as well as undermining cell competition by repressing Myc (discussed in detail in the ‘Role of feedback in *Drosophila* GSC niche robustness’ section) (Harris et al., 2011). Dpp/BMP signaling is also inhibited in these cells through the degradation of the Dpp/BMP receptor, Tkv (Xia et al., 2012).

### Refining long-range BMP gradients in the *Drosophila* wing imaginal disc

Dpp/BMP was first identified through studies of the *Drosophila* wing imaginal disc (Padgett et al., 1987; Spencer et al., 1982); therefore, it is not surprising that the wing disc has been a popular choice for investigation of Dpp/BMP gradient formation. Over the years, many studies have argued about the relative contribution of free diffusion and active mechanisms for Dpp/BMP gradient formation in this organ (reviewed by Akiyama and Gibson, 2015b; Kerszberg and Wolpert, 1998; Lander et al., 2002; reviewed by Müller et al., 2013; Ramírez-Weber and Kornberg, 1999; reviewed by Restrepo et al., 2014).

Dpp/BMP signaling in the *Drosophila* wing imaginal disc features a broad concentration gradient formed from secretion along a narrowly-expressed morphogen source (reviewed by Akiyama and Gibson, 2015b; Teleman and Cohen, 2000). Specifically, Dpp/BMP is expressed in a narrow stripe of anterior cells parallel to the anterior-posterior compartment boundary, forming a long-range morphogen gradient that regulates patterning and growth in both the anterior and posterior compartments of the wing disc (Affolter and Basler, 2007; Lecuit et al., 1996; Nellen et al., 1996) (Fig. 3). The long-range, 100 micron Dpp/BMP gradient of the *Drosophila* wing disc provides an instructive contrast to the 5 micron, single-cell-diameter gradient of the *Drosophila* germarium. However, as in the germarium, the spatial regulation of Dpp/BMP receptors and co-receptors by Dpp/BMP signaling itself is instrumental in regulating the range of the wing disc Dpp/BMP gradient (Lecuit et al., 1996; Norman et al., 2016).

In the wing disc, Dpp/BMP pathway activation induces transcriptional repression that decreases Tkv, Dally and Dally-like-protein (Dlp) levels in cells close to the morphogen source, allowing longer-range diffusion of the ligand (Crickmore and Mann, 2007; Fujise et al., 2003; Tanimoto et al., 2000). These cellular-level feedback mechanisms alone may not be sufficient for regulating the BMP gradient range, because local modulations of Dpp/BMP signaling would lead to cascading effects on the overall gradient. For example, a decrease in Dpp/BMP signaling at the periphery of the gradient would result in a compensatory increase in the local expression of Tkv and Dally. Increased receptor and co-receptor expression would increase local Dpp/BMP signaling, but also increase ligand sequestration, resulting in decreased diffusion across the wing disc. Recent work has characterized a secondary feedback loop in which another secreted factor Pentagone (Pent; also known as Magu) acts to internalize Dally and Dlp, thereby expanding the Dpp/BMP gradient (Hamaratoglu et al., 2011; Norman et al., 2016; Vuilleumier et al., 2010). This framework parallels the system in Box 2, in that Pent modifies the Dpp/BMP gradient in the wing disc by changing access to immobile binding sites. Pent is itself under negative Dpp/BMP regulation and therefore creates a coupled negative feedback loop that indirectly fine tunes the long-range Dpp/BMP gradient by modulating the balance between ligand release, trapping and receptor binding (Norman et al., 2016). The functional consequence of this nested feedback is discussed further in the ‘Role of feedback for scale-invariance during growth’ section.

## Tunability of BMP patterning through diffusible regulators

**Fig. 4.**
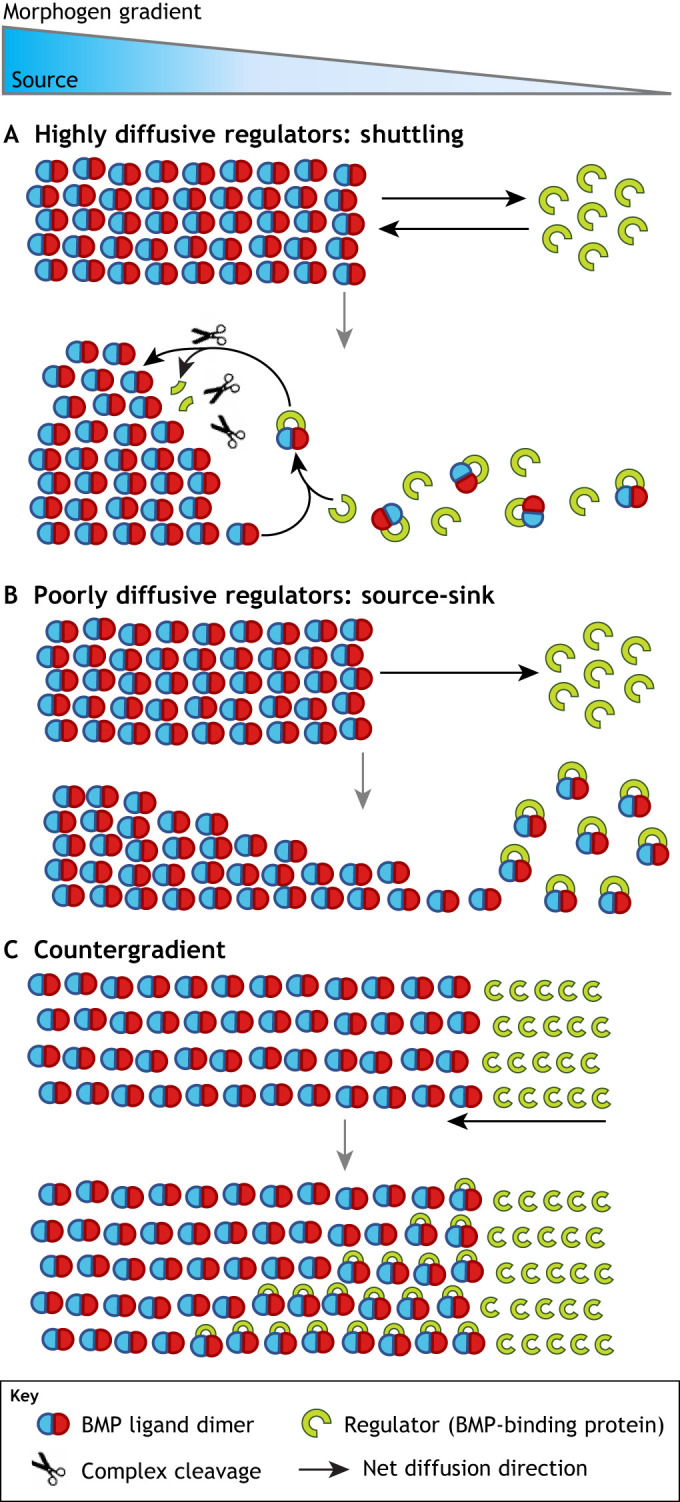
**Diversity of regulatory mechanisms.** (A) Highly mobile regulators can engage in shuttling processes, which have concentrating effects. Often ligand gradients end up smaller than their expression domain. Shuttling mechanisms establish peak signaling opposite of the regulator, regardless of where morphogen is expressed. (B) Poorly diffusive regulators have primarily inhibitory effects as they bind ligand and block signaling. This can act in a source-sink function as the ligand diffuses towards the ‘sink’ of immobile regulators. (C) Countergradients involve highly mobile regulators, as in shuttling. However, countergradient regulators do not have any pro-signaling functions.

All metazoan embryonic patterning systems feature a recurring cast of secreted, diffusible BMP-binding proteins that regulate BMP ligand gradient properties and pattern. However, the mechanistic roles played by these systems are very diverse, and several distinct models have emerged to explain them (Fig. 4). The primary regulator of BMP in embryonic dorsal-ventral (DV) patterning is the evolutionarily conserved BMP-binding protein Chordin. In the ‘shuttling model’, a highly mobile Chordin binds with high affinity to a poorly diffusive BMP ligand dimer and facilitates its diffusion (Barkai and Ben-Zvi, 2009; Ben-Zvi et al., 2014, 2011a, 2008; Holley et al., 1996). The conserved metalloprotease Tolloid cleaves Chordin in the BMP-Chordin complex, liberating the BMP ligand to either bind receptors or re-form a complex with Chordin (Fig. 4A). In the definitive shuttling model in the *Drosophila* embryo, this has the effect of concentrating the Dpp/BMP ligand away from the ventral expression domain of Sog (the *Drosophila* Chordin homolog), resulting in a Dpp/BMP ligand gradient narrower than the ligand expression domain.

In contrast to *Drosophila*, BMP gradient formation in the zebrafish embryo occurs through a ‘source-sink’ mechanism (Fig. 4B). In this model, a dorsally-expressed and restricted Chordin acts to bind and sequester BMP ligands dorsally, providing conditions for a net flux of BMP ligands towards the Chordin ‘sink’ and away from the BMP source (Reversade and De Robertis, 2005; Zinski et al., 2017). A third ‘countergradient’ model shares some features with the shuttling model, except that Chordin acts solely as a BMP antagonist to shape the BMP signaling gradient (Blitz et al., 2000; Connors et al., 1999; reviewed by Little and Mullins, 2006; Thomsen, 1997) (Fig. 4C). In this section, we illustrate these mechanisms along with other variations and combinations, as we describe how diffusible BMP inhibitors contribute to refining BMP gradients in the embryos of different species.

### A shuttling mechanism regulates the Dpp/BMP gradient in the *Drosophila* embryo

The *Drosophila* embryo is approximately 500 microns in length, with an average diameter of 180 microns. The Dpp/BMP signaling gradient starts broad, encompassing nearly half of the embryo's circumference, before forming a narrow, sharply peaked gradient spanning only five to seven cells (∼25-35 μm) at the dorsal midline (Dorfman and Shilo, 2001; Mizutani et al., 2005; O'Connor et al., 2006; Ross et al., 2001; Shimmi and O'Connor, 2003; Shimmi et al., 2005b; Umulis et al., 2006) (Fig. 1).

**Fig. 5.**
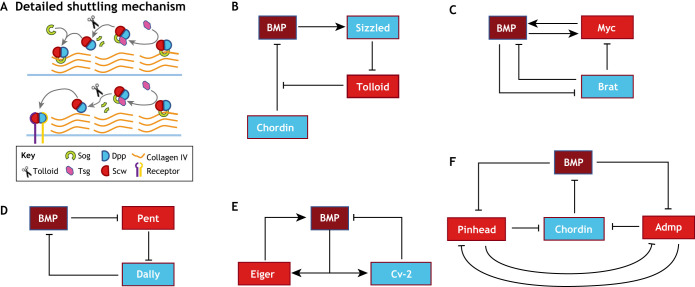
**Shuttling and extracellular regulation.** (A) BMP/Dpp shuttling as observed in *Drosophila* DV patterning. Sog binds Dpp/Scw ligand heterodimer and forms a Type IV Collagen bound complex that prevents signaling in areas of high Sog concentration. Tsg disrupts Sog-Dpp/Scw binding to Collagen and enables diffusion. Tsg also acts as a scaffold to promote Tolloid-mediated Sog cleavage and Dpp/Scw liberation. In areas with high Sog levels, the liberated ligand heterodimer typically reforms a Sog complex and begins another round of shuttling. In high Tolloid and low Sog levels areas, the ligand heterodimer is free to signal. Iterative rounds of complex formation and cleavage have the effect of moving ligand away from Sog expression, leading to a concentrated high peak at the dorsal midline. (B-F) Network diagrams of extracellular BMP regulation in diverse contexts. (B) BMP influences its own extracellular regulation in embryonic axis formation. BMP signaling leads to upregulation of the ventral protein Sizzled, which competitively inhibits Tolloid and prevents Tolloid-mediated Chordin cleavage. (C) In the *Drosophila* germarium, downstream BMP signaling target, Myc, provides positive feedback by upregulating BMP ligand uptake. Brat creates a bistable switch for differentiation by inhibiting BMP signal transduction and Myc activity. (D) In the *Drosophila* wing disc, Pent, a secreted factor that is negatively regulated by BMP signaling, supports BMP signaling by directing the internalization of Dally, a negative regulator of BMP signaling. (E) In the *Drosophila* embryo, downstream BMP signaling products Eiger and Cv-2 provide positive and negative feedback, respectively, to BMP signaling. These feedback mechanisms act to fine tune the BMP gradient and confer spatial bistability. (F) In zebrafish, Pinhead and Admp both act to support BMP signaling by promoting Chordin degradation. Pinhead and Admp are both downregulated by each other and by BMP signaling. The reciprocal repression circuit of Admp and Pinhead provides robustness in BMP gradient formation.

In *Drosophila*, *dpp* is expressed across the dorsal blastoderm, whereas its heterodimer partner ligand *scw* is expressed more broadly. A shuttling mechanism acts to concentrate the Dpp-Scw gradient into a sharp peak at the dorsal midline. The ventro-laterally expressed highly diffusive Sog binds the active Dpp-Scw heterodimer and prevents local receptor activation (François and Bier, 1995; O'Connor et al., 2006; Sawala et al., 2012; Shimmi et al., 2005b; Srinivasan et al., 2002; Umulis et al., 2009; Wang et al., 2008) (Fig. 4A). Cleavage of the Sog-Dpp/Scw complex by Tolloid liberates the Dpp/Scw heterodimer, allowing it to either bind to a receptor or form another complex with Sog. Iterative Sog binding facilitates diffusion of the ligand heterodimer away from high *sog* expression regions, and has the effect of concentrating the heterodimer at the dorsal midline at which Sog concentration is limited by the distance from its expression source and Tolloid cleavage (Shimmi et al., 2005b; Wang and Ferguson, 2005) (Fig. 5A).

This Sog-mediated shuttling mechanism has been further refined by experiments revealing the role of immobile ECM components (Wang et al., 2008). In Collagen IV-bound Dpp/Scw, the Scw ligand partially disrupts Collagen IV interaction with Sog and mediates the transfer of the Dpp/Scw heterodimer into a Collagen IV-Sog-Dpp/Scw complex. This complex interacts with a second Dpp/BMP-binding protein, Twisted gastrulation (Tsg), which mobilizes the shuttling complex by disrupting the remaining Sog-Collagen IV interaction. After release from Collagen IV, the shuttled complex (Tsg-Sog-Dpp/Scw) is able to diffuse freely, but unable to bind Dpp/BMP receptors until Tolloid-mediated cleavage of Sog (Shimmi et al., 2005b; Wang and Ferguson, 2005) (Fig. 5A).

Interestingly, Dpp/BMP-induced posterior crossvein (PCV) development in the *Drosophila* pupal wing appears to involve an adaptation of the shuttling mechanism for long-range signaling (Fig. 3). In this system, the Dpp/BMP ligand is expressed in longitudinal veins and Sog is expressed widely in the pupal wing, but both are notably absent from the PCV site (Ralston and Blair, 2005; Shimmi et al., 2005a). Other shuttling components, Tsg-paralog, crossveinless (Cv), and a Tld-paralog Tolloid-related (Tlr; Tok) are enriched in the PCV site (Matsuda and Shimmi, 2012; O'Connor et al., 2006; Serpe et al., 2005; Shimmi et al., 2005a). Therefore, long-range Dpp/BMP signaling from the longitudinal vein to the PCV may occur as Sog-Dpp complexes diffuse into the PCV site from all directions, where they are cleaved and liberated for signaling by Cv and Tlr.

### Mechanism in *Tribolium*: shuttling or source-sink?

Unlike *Drosophila*, in which *dpp* expression is limited to the dorsal blastoderm, in the flour beetle *Tribolium*, *dpp* is expressed uniformly along the putative DV axis (van der Zee et al., 2006). A broad *Tribolium* Dpp/BMP signaling gradient extends across the dorsal blastoderm, as observed through pMad activity (van der Zee et al., 2006) (Fig. 1). DV axis formation in *Drosophila* has diverged from many other patterning systems, whereas *Tribolium* is more representative of insects in terms of gene function and content (Richards et al., 2008; Van der Zee et al., 2008).

Relatively subtle differences in system components between *Tribolium* and *Drosophila* accommodate vastly different body plans and morphogen patterning processes. In *Tribolium*, Dpp gradient formation is not dependent on the intact Dpp-Sog shuttling mechanism observed in *Drosophila* (van der Zee et al., 2006). Genetic experiments indicate that *Tribolium* Tsg is not involved in shuttling, but is nevertheless required for Dpp/BMP signaling; suggesting a direct role for *Tribolium* Tsg, independent of Sog, in mediating Dpp/BMP binding to receptor (Nunes da Fonseca et al., 2010). In *Tribolium*, the function of Tolloid remains dependent on Sog, suggesting that its role in cleaving Sog from Dpp/Bmp is conserved between these two species (Nunes da Fonseca et al., 2010).

Interestingly, the broader Dpp/BMP signaling domain in *Tribolium* is reminiscent of the BMP signaling domain observed in vertebrate models, such as zebrafish and *Xenopus* (Fig. 1). In the *Drosophila* embryo, Dpp/BMP induces cell fates after rapid early gradient formation, whereas both vertebrate and *Tribolium* BMP gradients change dynamically during development. For example, in *Tribolium*, Dpp/BMP signaling-mediated cell fate specification occurs gradually as anterior cell fates are specified first (Nunes da Fonseca et al., 2010; Van der Zee et al., 2008). Similarly, the zebrafish BMP gradient is formed and maintained over several hours and specification occurs temporally along the anterior/posterior axis (Ramel and Hill, 2013; Tucker et al., 2008). In contrast, cell fates are specified within an hour in *Drosophila* (Sander, 1976).

The similarity in Dpp/BMP gradient profile between *Tribolium* and vertebrate species (Fig. 1) raises the possibility of similar underlying patterning mechanisms. Intriguingly, early DV patterning of *Tribolium* appears to be largely conserved from ancestral mechanisms shared with the spider *Achaearanea tepidariorum* (Akiyama-Oda and Oda, 2006; Nunes da Fonseca et al., 2010; reviewed by Wharton and Serpe, 2013) and other insects with broader Dpp/BMP gradients. Notably, *Drosophila* and other dipterans that share the more complex Tsg-Sog-Collagen shuttling mechanism have a sharply peaked Dpp/BMP signal at the dorsal midline, which specifies these cells to a unique extra-embryonic tissue called the amnioserosa (Panfilio, 2008; Rafiqi et al., 2008; Van der Zee et al., 2008). Perhaps the archetypal Tsg-Sog-Collagen shuttling mechanism observed in *Drosophila* embryogenesis is the result of adaptation to the unique biophysical constraints for amnioserosa formation. Indeed, this relationship underscores the significant role of Dpp/BMP signaling in the evolution of morphogenesis (Bier and De Robertis, 2015; Kwan et al., 2016).

Nevertheless, it is important not to overstate the similarities between the *Tribolium* and vertebrate systems. Although the BMP signaling gradient specifies similar cell fates in invertebrates and vertebrates (e.g. epidermis), the orientation of the DV axis itself and downstream organ development has been inverted in evolution (Arendt and Nübler-Jung, 1994). Thus, there is a ventral BMP gradient in vertebrates, as opposed to the invertebrate dorsal BMP gradient. Furthermore, although vertebrate models, such as zebrafish and *Xenopus* share the broader Dpp/BMP signaling domain of *Tribolium*, they also have spatially-defined ligand expression domains as seen in *Drosophila*.

## Diversity of gradient formation mechanisms by diffusible regulators in vertebrates

Although many of the molecular players are highly conserved, the vertebrate BMP signaling system diverges substantially from invertebrate systems (Arendt and Nübler-Jung, 1994; reviewed by Little and Mullins, 2006). In the *Xenopus* embryo, in which vertebrate BMP signaling was initially characterized, BMP gradient formation is driven by the complex spatiotemporal interactions of factors secreted by the dorsally-located Spemann organizer and a ventral pole (Fainsod et al., 1994; Re'em-Kalma et al., 1995; Schmidt et al., 1995). In this model system, dorsally-expressed Chordin, once again, acts as the primary antagonistic regulator of BMP; it is present at dramatically higher levels than BMP and binds the ligand to prevent receptor activation (Lee et al., 2006; Plouhinec et al., 2013). As in other species, the metalloprotease Tolloid cleaves both unbound and BMP-bound forms of Chordin, liberating BMP for receptor activation and downstream signaling (Larrain et al., 2001; Oelgeschläger et al., 2003). Layers of extracellular regulators modulate this central mechanism. A ventrally expressed factor, Tsg, provides both negative and positive regulation of BMP signaling; Tsg stabilizes the Chordin-BMP interaction by forming a Tsg-Chordin-BMP complex (Larrain et al., 2001; Lee et al., 2006; Ross et al., 2001), but also demonstrates pro-BMP activity by scaffolding Tolloid-mediated Chordin cleavage (Larrain et al., 2001; Oelgeschläger et al., 2003; Scott et al., 2001). Regulation of Tolloid function is another major avenue of BMP regulation; Ont1 (Olfml3) also acts to scaffold Tolloid-Chordin cleavage, and ventral proteins Sizzled and Crescent competitively inhibit Tolloid (Fig. 5B) (Inomata et al., 2008; Lee et al., 2006; Ploper et al., 2011). Free BMPs also non-competitively inhibit Tolloid activity (Lee et al., 2009).

Noggin and Follistatin (reviewed by Little and Mullins, 2006), work similarly to Chordin by directly binding to BMP ligands. However, they are not cleaved by Tolloid processing and primarily function to block BMP signaling in the dorsal organizer (Dal-Pra et al., 2006; Khokha et al., 2005). Additional secondary regulators of BMP signaling are discussed in the context of feedback-mediated adaptability and robustness later.

Generating a mechanistic understanding of these complex spatiotemporal interactions is a daunting task. To date, *Xenopus* studies have largely advanced a Chordin countergradient theory of BMP gradient formation with shuttling; however, some studies argue against the involvement of shuttling by showing that *chordin* morphants have no change in ventral BMP signaling (Ben-Zvi et al., 2014; Francois et al., 2009; Plouhinec et al., 2013), and Chordin acts at short-range (Blitz et al., 2000). Integrated computational and quantitative biophysics approaches are needed to clarify the patterning mechanism and investigate the viability of alternatives, such as the source-sink mechanism.

In the zebrafish embryo, shuttling (Zhang et al., 2007), source-sink, countergradient (Blitz et al., 2000; Connors et al., 1999; Thomsen, 1997) and transcriptional models (in which the BMP ligand is relatively immobile and its signaling is dictated by the BMP expression domain; Ramel and Hill, 2013), have all been proposed as mechanisms for BMP gradient formation. Recently, quantitative measurements of biophysical properties and large-scale computational screening of biophysical parameters have been used to test mechanisms of BMP gradient formation (Pomreinke et al., 2017; Zinski et al., 2017). These results suggested a source-sink mechanism for zebrafish embryo BMP gradient formation that emerges from diffusible BMP ligands and Chordin acting as a dorsal sink for the BMP ligand (Zinski et al., 2017). Further elaborating on this mechanism are a series of recent molecular-genetic experiments showing that the Tolloid/Bmp1a metalloprotease homologs effectively restrict Chordin to dorsal regions, preventing it from diffusing into ventral regions (Tuazon et al., 2020). Directly testing the role of Chordin as a dorsal sink, a membrane-tethered Chordin in a background lacking the metalloproteases and endogenous Chordin was shown, remarkably, to rescue DV patterning (Tuazon et al., 2020). Computational modeling of immobile Chordin supports gradient rescue for a large number of solutions that simulate the experiment with localized expression of lateral and dorsal membrane-tethered Chordin (Tuazon et al., 2020).

In contrast, computer simulations suggest that a highly diffusive Chordin and a highly selective Tolloid cleavage of BMP-Chordin would be required to achieve a steep *Drosophila*-like BMP gradient in zebrafish (Zinski et al., 2017). In a different set of experiments, it was found that making *Drosophila* Sog more Chordin-like by modification, so that it is cleaved by Tolloid independent of BMP binding, creates a wider and shallower BMP signaling profile in *Drosophila* that is more reminiscent of the BMP gradients in *Tribolium* and zebrafish (Peluso et al., 2011).

Further variations in the Chordin-BMP relationship can be found in less-studied invertebrate species. For example, in the sea urchin, Chordin and BMP are co-expressed ventrally (Lapraz et al., 2009). In this system, Chordin is responsible for spatial restriction of BMP, but is not required for long-range BMP diffusion to the dorsal region. Studies in the *Nematostella* (sea anemone) embryo also found co-localization of Chordin and BMP expression domains (Genikhovich et al., 2015). Interestingly, computational studies of the location of BMP and Chordin expression domains suggest that the spatial positioning of Chordin expression, but not BMP, is determinative for gradient formation; in shuttling systems the BMP signaling peak is located opposite the Chordin expression domain regardless of the BMP expression domain (Genikhovich et al., 2015). Indeed, *Drosophila* experiments show that the BMP gradient location is defined solely by Sog expression (Wang and Ferguson, 2005). These results highlight the versatility of a small set of regulators to produce BMP gradients adapted to diverse contexts through subtle biophysical modifications.

## BMP morphogen system: feedback-mediated adaptability and robustness

The plasticity of BMP signaling in adapting to diverse length scales across developmental contexts belies remarkable robustness within each system in response to genetic and environmental perturbations. The dynamic regulation of the range and availability of BMP in normal and experimentally perturbed systems provides a glimpse into how gradient shape and range are highly tunable for diverse patterning objectives.

### Role of feedback in *Drosophila* GSC niche robustness

A closer look at the *Drosophila* germarium reveals that feedback mechanisms not only support intercellular Dpp/BMP signal interpretation (discussed above), but also confer robustness to the extracellular Dpp/BMP gradient itself (reviewed by Harris and Ashe, 2011; Harris et al., 2011). Cell-competition within the GSC niche is largely mediated by Myc, a transcription factor highly expressed in GSC cells, that supports increased overall protein synthesis and enhanced ligand uptake (Harris et al., 2011; Liu et al., 2015; Moreno and Basler, 2004; Rhiner et al., 2009). The ligand-clearance function of Myc is crucial in regulating the scale of the Dpp/BMP gradient; experimental situations in which Myc-expressing GSCs are removed from the niche causes expansion of the Dpp/BMP gradient further into the germarium (Harris et al., 2011; Kai and Spradling, 2004; Liu et al., 2015; Rhiner et al., 2009). Under these conditions, increased Dpp/BMP availability in cystoblasts triggers a Myc-mediated feedback loop that drives cell competition, ultimately leading to dedifferentiation of developing cyst cells (Fig. 5C) (Harris et al., 2011). The newly de-differentiated cell reoccupies the niche and resumes the spatial restriction of the Dpp/BMP gradient, presumably by expressing molecules that bind and titrate the ligand. Thereafter Myc-mediated ligand clearance resumes spatial regulation of the Dpp/BMP gradient scale (Box 2).

Computational modeling approaches indicate that it is specifically the ligand-uptake effects of Myc that mediate its role in differentiation and cell competition between GSCs and cystoblasts (Harris et al., 2011). The discovery of the Brat-mediated mechanism for stem cell differentiation and gradient robustness was aided by the application of model-based design-of-experiments (MBDOE) and multiobjective optimization to integrate disparate qualitative datasets and identify a limited number of parsimonious regulatory networks consistent with published data (Harris et al., 2011; Pargett et al., 2014).

### Feedback and scaling in embryo development

Scale invariance, the maintenance of a consistent pattern at different sizes, is a hallmark of Dpp/BMP signaling in embryo development and highlights the robustness of the system. In the *Drosophila* embryo, dorsal surface patterning by Dpp/BMP exhibits scaling between closely related species and between individuals within a species (Umulis et al., 2010). Specifically, the ratio of average Dpp/BMP-induced pMad pattern width-to-embryo length is constant between *Drosophila melanogaster* and related species, the larger *Drosophila virilis*, and the smaller *Drosophila busckii.* Individual embryos of differing sizes within each of those species maintain this constant ratio as well (Umulis et al., 2010). More recently, the zebrafish embryo has been reported to maintain scaling of the Dpp/BMP signaling gradient in the face of experimental reductions in embryo size of up to 30% (Huang and Umulis, 2019). Computational and experimental studies in *Xenopus* have determined that the Chordin gradient, and specifically the mechanism of Sizzled-regulated Tolloid cleavage of Chordin, is required for BMP signaling scale invariance (Ben-Zvi et al., 2008; Inomata et al., 2013). Understanding the mechanisms of, and requirements for, scale invariance is valuable for understanding BMP-mediated pattern formation. As an example of biologically achieved robustness, scale invariance demonstrates a selective advantage of BMP systems. Including this ‘performance’ objective as a metric in multi-objective optimization approaches can aid in evaluating competing mechanistic models of BMP gradient formation.

### Role of feedback for scale-invariance during growth

In addition to interspecies and intraspecies scale invariance, BMP gradients also exhibit scaling within a growing domain or ‘dynamic scaling’. This phenomenon has most famously been characterized in the *Drosophila* wing imaginal disc, in which the amplitude of the Dpp/BMP gradient has been shown to dynamically scale with disc growth (Fried and Iber, 2014; Harmansa et al., 2015; Wartlick et al., 2011). Modeling work suggests multiple potential mechanisms for scaling in the wing disc (Ben-Zvi and Barkai, 2010; Ben-Zvi et al., 2011b; reviewed by Hamaratoglu et al., 2014; Umulis, 2009; Umulis and Othmer, 2013), including advection of cell-bound ligand (Fried and Iber, 2014), pre-steady state diffusion (Fried and Iber, 2014) and modulation of effective diffusion rates via concentration of extracellular regulators (Ben-Zvi and Barkai, 2010; Umulis and Othmer, 2013).

The advection model describes a simple mechanism in which the morphogen gradient is scaled as ligand is carried away from the source by growing cells. Advection necessarily contributes to gradient formation and scaling, but its relative contribution may be limited in the wing disc as the relatively small growth at the ligand source favors diffusion (Fried and Iber, 2014). The pre-steady state diffusion model describes scaling as a natural consequence of morphogen diffusion (Fried and Iber, 2014). This model requires that tissue growth is substantially faster than diffusion to prevent the morphogen from equilibrating into a steady state across the domain. In addition, a pre-steady state diffusion model makes testable hypotheses about the decay rate – the Dpp/BMP half-life must be at least 10 and more likely 48 h (Fried and Iber, 2014). It remains unclear whether this is a viable ligand decay rate in the wing imaginal disc (Kicheva et al., 2007; Teleman and Cohen, 2000; Wartlick et al., 2011).

Interestingly, the pre-steady state diffusion model argues that morphogen gradient scaling and domain growth can be independent processes. Indeed, recent experimental studies using conditional knockouts have shown that the Dpp/BMP stripe in third instar larvae is crucial for patterning, but not for wing disc growth (Akiyama and Gibson, 2015a). In contrast, more recent work using two conditional *dpp* alleles indicates that, although the Dpp stripe is essential for wing disc growth, graded BMP signaling is not (Barrio and Milán, 2017; Bosch et al., 2017; Harmansa et al., 2015; Matsuda and Affolter, 2017). That is, a minimal threshold of Dpp/BMP signaling is needed for growth, but this is a distinct mode of action from Dpp/BMP gradient-induced patterning. Investigating the relationship between BMP-regulated patterning and growth is crucial for understanding BMP signaling system function.

It has been proposed that feedback from extracellular regulators can produce dynamic scaling through an ‘expansion-repression’ mechanism (Ben-Zvi and Barkai, 2010). In this model, an ‘expander’ molecule acts to support the effective morphogen diffusion rate either by directly facilitating diffusion or by inhibiting degradation. The expansion activity is tied to tissue growth, providing scaling, through negative regulation or ‘repression’ by the morphogen itself. In the imaginal wing disc, Pent, which is under negative feedback regulation by Dpp/BMP and acts to expand the Dpp/BMP gradient via downregulation of Dpp/BMP inhibitors, has been suggested as an expander in an expansion-repression mechanism that scales the Dpp/BMP gradient (Fig. 5D) (Ben-Zvi and Barkai, 2010; Ben-Zvi et al., 2011a). The expansion-repression mechanism has also been suggested as the mechanism underlying scale invariance in the zebrafish pectoral fin. In that system, Smoc1, a conserved secreted factor, supports BMP signaling in an expander role analogous to Pent in the wing disc (Mateus et al., 2020). More recent work from Zhu and colleagues questions whether Pent acts as an ‘expander’ in the expander-repressor model due to its limited spatial range (Zhu et al., 2020). Their work proposes an alternative model in which the role of Pent as an expander is limited to the very early stages of wing disc growth. Instead, a pseudo source-sink mechanism of morphogen-mediated regulation of receptor function is primarily responsible for scaling through most of wing-disc growth.

### Determining the functional consequence of feedback in other BMP systems in development

Recent work on the Nodal TGF-β ligand and its feedback inhibitor Lefty shows that development and patterning can be fully rescued in *lefty* mutants without restoring the feedback mechanism. However, rescued *lefty* zebrafish mutants remain less tolerant of mild perturbations in Nodal signaling levels, indicating that patterning without inhibitory feedback is functional but fragile (Rogers et al., 2017). Related studies are needed to better determine robustness and fragility of BMP systems with hindered feedback. Intriguingly, recent modeling work in the *Drosophila* wing imaginal disc indicates that cytonemes may allow for gradient formation without the addition of extrinsic noise, suggesting a potential division of labor between cytoneme- and diffusion-based mechanisms, depending on the noise sensitivity of a given patterning niche (Fancher and Mugler, 2020). Perhaps the prevalence and complexity of feedback loops in a given patterning niche may be indicative of the relative role of diffusion and cytonemes in gradient formation.

In our preceding examples, morphogen patterning that is regulated through intricate feedback mechanisms leads to robustness and this can confound identification of developmental mechanisms through genetic analysis. Networks with feedback can often compensate for perturbations, including the loss or partial loss of a factor in the network, the loss of cells or changes in embryo size by the upregulation or downregulation of compensatory components and pattern remodeling.

A striking example of this phenomenon is provided by the *Drosophila* embryo, in which the shuttling mechanism of gradient formation is refined by the action of Eiger (Egr), a homolog of TNF-α, and Crossveinless 2 (Cv-2), a membrane-bound Dpp/BMP regulator. Egr provides positive feedback regulation to Dpp/BMP signaling through the JNK pathway. Cv-2 has been shown to both positively and negatively regulate Dpp/BMP signaling (Fig. 5E) (Binnerts et al., 2004; Coles et al., 2004; Conley et al., 2000; Ikeya et al., 2006; Kamimura et al., 2004; Rentzsch et al., 2006; Zhang et al., 2010; Zhang et al., 2008). Computational modeling approaches have integrated the disparate experimental data to reveal that low Cv-2 levels promote BMP signaling, whereas high Cv-2 expression levels inhibit Dpp/BMP signaling by sequestering ligand dimers and preventing receptor activation (Serpe et al., 2008). Together, the coupled positive and negative feedback of Egr and Cv-2 confer spatial bistability to the Dpp/BMP gradient and are crucial to the characteristic peak of Dpp/BMP signaling at the dorsal midline. The concentration-specific effect of Cv-2 has suggested a potential role in Dpp/BMP signaling noise reduction (Karim et al., 2012). This computational prediction is seemingly opposed by the minimal increases in Dpp/BMP signaling variability observed in *Drosophila cv-2* mutants. However, disruption of Cv-2 and Egr in *cv-2; egr* double mutants shows substantially increased BMP signaling intensity and variability (Gavin-Smyth et al., 2013).

In a zebrafish example, anti-dorsalizing morphogenetic protein (Admp) and Pinhead have been identified as another set of coupled regulators of BMP signaling (Fig. 5F) (Yan et al., 2019). Admp is a dorsally expressed BMP-like protein that binds and promotes the degradation of Chordin, and is transcriptionally repressed by BMP signaling (Reversade and De Robertis, 2005; Yan et al., 2019). As with Egr and Cv-2, Admp mutants have reported only minimal phenotypes. Pinhead is a newly discovered BMP-like protein which, like Admp, can promote Chordin degradation. In addition, like *admp*, *pinhead* loss-of-function mutants show only minimal dorsalization of the BMP signaling gradient. However, *pinhead* mutants were observed to have increased *admp* expression and *admp* mutants increased *pinhead* expression, demonstrating their reciprocal repression and potential compensatory regulation. Supporting their compensatory functions, double mutants of *admp* and *pinhead* exhibit a strongly dorsalized phenotype, contrasting the weak single mutant phenotypes. Given their redundant functions possibly as scaffolds for Chordin degradation, the reciprocal repression circuit between these two proteins allows each protein to compensate for the absence of the other. Together the Egr-Cv2 and Admp-Pinhead examples illustrate how the lack of a phenotype can mask a sensitized and fragile system, as redundancy and feedback effects mitigate the impact of single loss-of-function mutants.

## Future perspectives

The first quarter century of research on the BMP signaling system identified a recurring cast of regulators that shape BMP gradients in a variety of developmental contexts. However, biochemistry and genetics approaches proved limited in generating mechanistic understanding of BMP gradient formation in different systems, as it became increasingly apparent that multiple gradient formation mechanisms are viable given different biophysical properties or regulatory networks. In recent years, new approaches that integrate genetics, embryo perturbations, imaging and high-throughput computational modeling have begun to clarify the complex interplay between biochemical networks that include feedback mechanisms that regulate BMP-mediated morphogen patterning. Currently, most studies of the BMP system evaluate hypothesized mechanisms, either experimentally or computationally, based on their ability to explain the morphogen gradient signaling distribution. This approach may be overlooking several important properties of BMP patterning that could further constrain models and identify underlying regulatory principles.

In this Review, we have described scale invariance and noise reduction as additional lenses for investigating the robustness of the BMP signaling system. Understanding dynamic changes in the BMP signaling in three-dimensional space over developmental timescales presents another intriguing avenue for future research. In fact, mammalian systems, which have been understudied, have longer developmental periods and greater roles for redundant BMP antagonists (Bier and De Robertis, 2015). Therefore, understanding the role of tertiary regulators may prove to have more relevance to human disease than the core network that has primarily been studied to date. For example, in mouse studies, Noggin has been identified as a key regulator in the formation of the neurogenic border of the olfactory system (Forni et al., 2013), as well as in neural tube formation and axial skeletal formation (Brunet et al., 1998; McMahon et al., 1998; Wijgerde et al., 2005). An intriguing recent study in the mouse embryo suggests a significant role for embryo geometry in BMP gradient formation. Specifically, the basolateral localization of BMP receptors in the early mouse embryo is required to protect against morphogen signaling fluctuations and preserve robust BMP gradient formation (Zhang et al., 2019). A recent 4D model of zebrafish BMP patterning through epiboly provides a template for fully incorporating spatial and temporal considerations in computational studies of the BMP system (Li et al., 2019). Adding a requirement for patterning in higher dimensions allows additional data to be considered that can further constrain simulations and further refine models without additional experimental burden. Many of the embryos and tissues discussed herein are imaged *in toto* and thus the data are already at hand.

As the field has moved from identifying and characterizing the components that interact to form the gradients towards deciphering how the molecules all work in concert as a reliable and robust system, there will continue to be an increasing need for ways to combine the data into mathematical models of the processes. These models should grow to become more user-friendly as a simulation-based aid for testing alternative hypotheses and predicting how a planned experiment may impact robustness, fragility or other emergent behavior. Similarly, as more is known about the limits to a biological system under diverse perturbations, these new data need to be better utilized for identifying the most consistent mathematical models. Multi-objective optimization, MBDOE and large-scale computational screens will be necessary to integrate data from disparate model systems, to generate holistic understanding of the biological mechanisms at play and to identify experiments to expose underlying fragility once key regulatory steps are removed. Developmental biology in general, and the BMP field in particular, has long been at the forefront in integrating systems and engineering approaches to address biological questions. A renewed focus on generating computational methods accessible for all developmental biologists will pay dividends in increasing the pace of scientific progress in understanding development.
